# New‐Onset Complex Partial Seizures Progressing to Intractable Epilepsy in a Young Female With Bifrontal Encephalomalacia and a Remote History of Traumatic Brain Injury

**DOI:** 10.1002/ccr3.71830

**Published:** 2026-01-11

**Authors:** Katrina J. Villegas, Aqsa Sorathia, Marina Salib, Jessica Escobar, Nicole Conroy

**Affiliations:** ^1^ Department of Internal Medicine St. Joseph's University Medical Center Paterson New Jersey USA; ^2^ Health Sciences Library St. Joseph's University Medical Center Paterson New Jersey USA; ^3^ Department of Neurology St. Joseph's University Medical Center Paterson New Jersey USA

**Keywords:** complex partial seizure, encephalomalacia, frontal lobe epilepsy, intractable epilepsy

## Abstract

Encephalomalacia, the irreversible loss of brain tissue following injury, is a well‐documented cause of post‐traumatic epilepsy, typically manifesting within the first year. However, delayed‐onset intractable epilepsy in young adults remains underrecognized. We present a case of a young female with bifrontal encephalomalacia secondary to a traumatic brain injury (TBI) six years prior who developed complex partial seizures progressing to intractable epilepsy. Electroencephalography (EEG) confirmed seizure activity predominantly from the right frontal lobe. Despite multiple anti‐seizure medications, the patient required intensive care management for refractory status epilepticus. This case highlights the potential for chronic epileptogenesis in TBI survivors, emphasizing the need for long‐term vigilance, strict medication adherence, and proactive follow‐up to optimize seizure control and prevent recurrence.

## Key Clinical Message

Post‐traumatic encephalomalacia is an underrecognized cause of delayed‐onset epilepsy in young adults. This case highlights a young female who developed intractable epilepsy six years after a minor TBI, attributed to bifrontal encephalomalacia. The progression underscores the need for early diagnosis, strict medication adherence, and long‐term follow‐up in TBI patients.

## 1 Introduction

Post‐traumatic epilepsy is a well‐recognized complication of traumatic brain injury (TBI), typically manifesting within the first year [1, 2, 3]. However, delayed‐onset epilepsy may occur years after, particularly in cases where structural brain damage, such as encephalomalacia, is involved [3]. Although delayed‐onset epilepsy in young adults is rare, it poses significant challenges in both diagnosis and management.

We present a rare case of a young adult who developed intractable epilepsy that emerged six years after a seemingly minor TBI, with bifrontal encephalomalacia identified as the underlying pathology. The purpose of this report is to emphasize the need for vigilance in managing patients with a history of TBI, even when initial presentations appear benign. Furthermore, it demonstrates the diagnostic and therapeutic strategies employed in addressing intractable epilepsy associated with remote TBI‐induced encephalomalacia. Early recognition, strict adherence to anti‐seizure medications, and long‐term follow‐up are critical to optimizing patient outcomes and minimizing the risk of refractory epilepsy.

## 2 Case History/Examination

A 25‐year‐old female presented with acute neurobehavioral changes, described as episodes of screaming and non‐responsiveness to conversations that had progressively increased in frequency over two days. One month prior, she experienced a generalized tonic–clonic seizure, where she was informed of a “frontal lobe abnormality” on prior imaging. She was prescribed levetiracetam 1000 mg twice daily, but reported intermittent adherence. There was no childhood history of epilepsy, recent head trauma, or other neurologic complaints, but she recalled a minor head injury six years earlier involving impact against a hard surface without loss of consciousness or subsequent medical evaluation.

**FIGURE 1:**
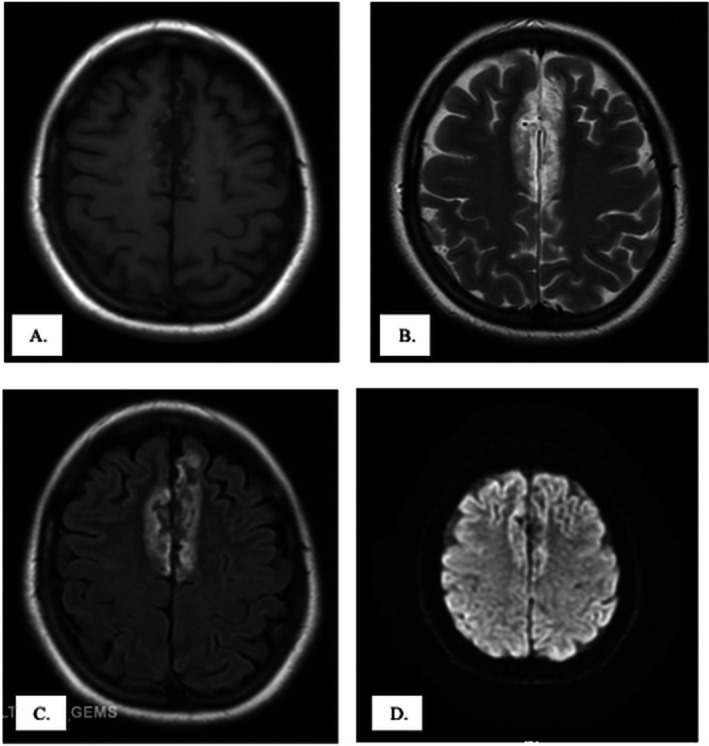
MRI of the brain showing bilateral frontal lobe lesions consistent with Bifrontal Encephalomalacia in different MRI sequences. T1‐weighted image (A) and Diffusion‐Weighted image (DWI, D) show hypointense bifrontal focal lesions. T2‐weighted image (B) and Fluid‐attenuated inversion recovery image (FLAIR, C) both show hyperintense bifrontal focal lesions. These findings reflect chronic post‐traumatic structural brain damage, which served as the epileptogenic substrate for the patient's delayed‐onset seizures. Identifying such lesions is critical in patients with remote head trauma, as they may predict long‐term risk of post‐traumatic epilepsy.

In the emergency department, examination revealed intermittent word‐finding difficulties and incomprehensible vocalizations accompanied by self‐limited left lower lip twitching. CT scan of the brain without contrast showed frontal lobe encephalomalacia. Brain MRI demonstrated bilateral medial frontal lobe encephalomalacia (Figure 1). The patient was diagnosed with trauma‐induced encephalomalacia‐associated epilepsy and admitted for continuous electroencephalography (EEG) monitoring and medical optimization.

**FIGURE 2:**
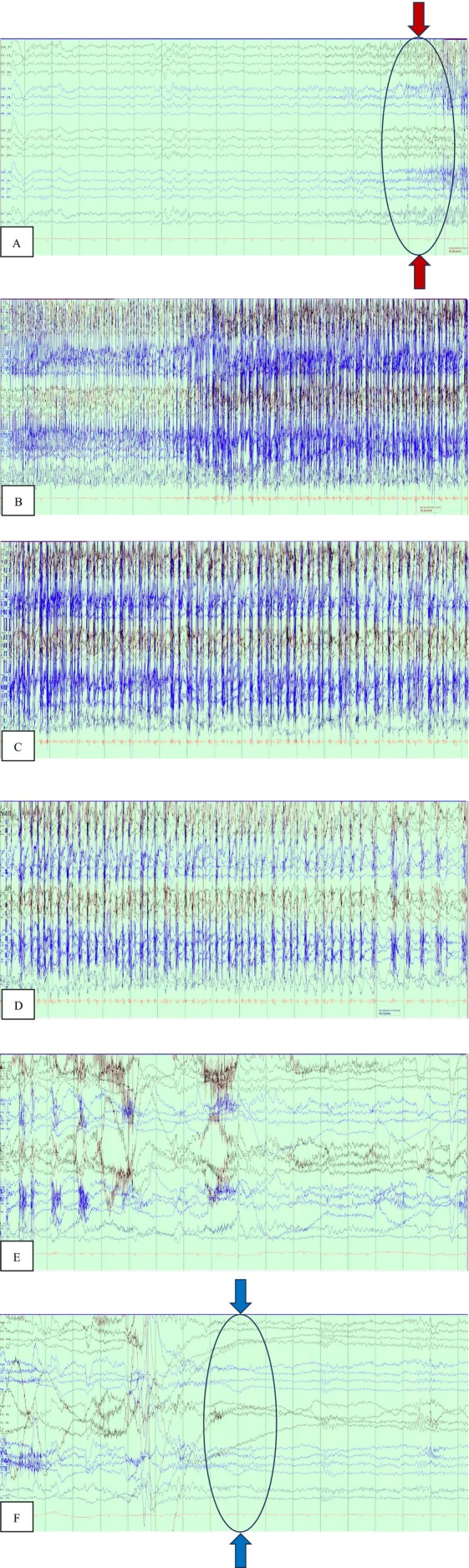
Video Electroencephalography (EEG) 24‐h recording demonstrates the evolution of a focal seizure. Panel A shows a normal EEG background prior to seizure onset. Progressive development of epileptiform activity is evident in Panels B–D, characterized by sharp waves and spike discharges (*red arrows and encircled area mark the onset of epileptiform activity*). The discharge culminates in a clinical seizure event before resolving and returning to baseline, as shown in Panels E, F (*blue arrows and encircled area indicate the end of the epileptiform discharges*). These findings correlate with the patient's motor automatisms and focal seizures, confirming the frontal lobe epilepsy secondary to bifrontal encephalomalacia. Continuous EEG monitoring was essential for establishing the diagnosis and guiding escalation of anti‐seizure therapy.

During hospitalization, she exhibited episodic involuntary facial twitching and rhythmic left upper extremity movements, occasionally accompanied by gaze deviation and transient speech impairment. These focal seizures evolved into clusters occurring every few minutes, each lasting less than a minute and followed by mild postictal confusion and irritability, without loss of consciousness or incontinence. Video EEG 24‐h recording demonstrated focal epileptiform discharges predominantly from the right hemisphere, with occasional contralateral activity, correlating with the observed semiology and confirming the diagnosis of frontal lobe epilepsy (Figure 2).

The patient experienced recurrent focal seizures despite dual anti‐seizure therapy with fosphenytoin and levetiracetam. A subtherapeutic phenytoin level prompted escalation of therapy, including additional lorazepam, valproic acid, and adjunctive topiramate. Ongoing electrographic seizures necessitated transfer to the medical intensive care unit for refractory status epilepticus, requiring intubation, sedation with midazolam infusion, and continuous EEG monitoring. Seizure activity resolved after seven days, confirmed by three consecutive seizure‐free EEG recordings. The patient was subsequently extubated and transitioned to oral anti‐seizure medications phenytoin, levetiracetam, and topiramate before discharge on hospital day 15.

Two months later, she was re‐admitted for breakthrough seizures following two days of medication nonadherence. Neuroimaging showed no new findings compared with prior studies, and her phenytoin level was again subtherapeutic. After reinitiation of intravenous fosphenytoin, levetiracetam, and topiramate, seizure control was re‐established. She was counseled extensively on strict adherence to her medication regimen and discharged on oral levetiracetam 1500 mg twice daily and topiramate 100 mg twice daily.

At outpatient follow‐up two weeks later, the patient reported full compliance with therapy and no seizure recurrence. She demonstrated good tolerance to her medications without adverse effects. Ongoing management focused on continued pharmacologic therapy, psychosocial support, and education emphasizing adherence and long‐term follow‐up to mitigate the risk of recurrence and optimize quality of life.

## 3 Methods

The patient underwent comprehensive clinical evaluation, neuroimaging with CT and MRI, and continuous EEG monitoring to establish the diagnosis of frontal lobe epilepsy secondary to bifrontal encephalomalacia. The diagnostic approach prioritized structural and electrographic correlation to exclude psychogenic etiologies and guide subsequent management.

## 4 Discussion

Encephalomalacia refers to the irreversible loss of brain parenchyma following injury and represents a recognized substrate for epileptogenesis. Although most cases are described in pediatric and geriatric populations, adult‐onset post‐traumatic encephalomalacia remains underreported [1]. Neurologic manifestations commonly include focal seizures [4, 5], and MRI plays a crucial role in its diagnosis by demonstrating chronic parenchymal loss with fluid‐attenuated inversion recovery (FLAIR) hyperintensities, often corresponding to epileptogenic foci on EEG [6].

Post‐traumatic epilepsy develops in approximately 5% of patients with prior traumatic brain injury (TBI) and may manifest years after the initial insult, particularly in cases involving structural cortical damage such as encephalomalacia [2, 7, 8, 9]. The pathophysiology involves chronic neuronal hyperexcitability, gliosis, chronic neuroinflammation, and maladaptive synaptic reorganization that disrupt inhibitory pathways, leading to spontaneous seizure generation [10, 11]. These processes may persist for years, explaining the delayed onset observed in this patient six years post‐TBI.

Management of epilepsy secondary to encephalomalacia parallels that of other structural epilepsies, although achieving seizure control can be challenging. Polytherapy with synergistic anti‐seizure medications (ASMs) is frequently required, as monotherapy often proves insufficient in the setting of focal cortical injury [12, 13, 14]. In this case, escalation from dual therapy due to a multidrug regimen was necessary to achieve remission of status epilepticus.

Surgical intervention may be considered in refractory cases when a well‐localized epileptogenic focus is amenable to resection. However, bilateral encephalomalacic lesions and multifocal epileptiform discharges, as observed in this patient, limit the feasibility and potential benefit of surgical approaches [15, 16, 17]. Neuromodulation techniques, including vagus nerve or responsive neurostimulation, remain alternatives but are best evaluated once the patient achieves medical stability [3, 8].

Long‐term management should emphasize medication adherence, psychosocial support, and consistent neurologic follow‐up. Nonadherence remains a major contributor to seizure recurrence and hospitalization. Comprehensive counseling, patient education, and integration of mental health and social support services are vital to improving compliance and overall quality of life in individuals with post‐traumatic encephalomalacia.

## 5 Conclusion and Results

This case illustrates delayed‐onset intractable epilepsy arising from bifrontal encephalomalacia six years after mild traumatic brain injury (TBI). Management requires long‐term vigilance, strict adherence to anti‐seizure medications, and proactive follow‐up. While surgery may benefit select patients with focal lesions, bilateral involvement often necessitates chronic medical therapy. Clinicians should remain alert to the potential for late epileptogenesis in TBI survivors.

## Author Contributions

**Katrina J. Villegas:** conceptualization, data curation, formal analysis, visualization, writing – original draft, writing – review and editing. **Aqsa Sorathia:** writing – original draft, writing – review and editing. **Marina Salib:** supervision. **Jessica Escobar:** resources. **Nicole Conroy:** resources, supervision.

## Funding

The authors have nothing to report.

## Ethics Statement

An ethical review is not necessary because this is a clinical case report.

## Consent

As this is a clinical case report, written consent was obtained for the purpose of this paper.

## Conflicts of Interest

The authors declare no conflcits of interest.

## Data Availability

The data that support the findings of this study are available from the corresponding author upon reasonable request.
